# Epigenetic strategies for mitigating kidney fibrosis

**DOI:** 10.7717/peerj.21709

**Published:** 2026-09-14

**Authors:** Cong Wang, Yuntong Wu, Lijuan Yu

**Affiliations:** 1 Department of Pharmacy, Lu’an People’s Hospital of Anhui Medical University, Lu’an, China; 2 Department of Rehabilitation, Lu’an People’s Hospital of Anhui Medical University, Lu’an, China

**Keywords:** Renal fibrosis, Epigenetic, DNA methylation, Histone modifications, Non-coding RNAs

## Abstract

Renal fibrosis is the irreversible pathological core of chronic kidney disease (CKD). Current treatments can slow disease progression, but cannot cure it. Epigenetic regulatory mechanisms provide new perspectives for understanding and intervening in this process. This review summarizes the core epigenetic regulatory networks involved in renal fibrosis, including DNA methylation, histone modification, and non-coding RNA regulation. First, abnormal DNA methylation can silence antifibrotic genes. Second, histone modifications dynamically regulate the chromatin state and affect the transcription of fibrosis-related genes. Moreover, ncRNAs regulate fibrosis signaling through complex networks. Preclinical studies support the potential value of DNA methyltransferase (DNMT) inhibitors, histone deacetylase (HDAC) inhibitors, micro RNA-based strategies, N6-methyladenosine (m6A)-targeted approaches, natural products, and programmable epigenetic editing. Several barriers limit clinical translation. Future developments will depend on precision medicine. Optimizing targeted therapies, designing rational combination therapies, and establishing a strict, systematic long-term safety assessment system are key to translating epigenetic research results into effective treatments for renal fibrosis.

## Introduction

Renal fibrosis is a common histopathological feature of chronic kidney disease (CKD) and is strongly linked to progression toward kidney failure (Abbad, Esteve & Chatziantoniou, 2025). It is formed by repeated tubular epithelial injury, persistent inflammation, macrophage activation, myofibroblast expansion, vascular rarefaction, metabolic stress, oxidative stress, ferroptosis, and abnormal extracellular matrix (ECM) deposition (Huang, Fu & Ma, 2023). This pathological scar disrupts the structure of the kidneys, affects over 10% of adults worldwide and causes significant clinical and economic burdens (Abbad, Esteve & Chatziantoniou, 2025)**.** According to the 2021 Global Burden of Disease Study, CKD is the 11th leading cause of death globally, and the number of years of life lost because of health impairments caused by renal dysfunction ranks 8th (Deng et al., 2025). Data from China show that the prevalence of CKD is as high as 8.2% and that is incidence and prevalence are expected to continue to increase over the next decade (Guo et al., 2024). This difficult epidemiological situation underscores the urgency of curbing CKD progression.

However, the clinical treatment needs for the core aspects of kidney fibrosis have not been fully met. Current treatment strategies mainly focus on controlling the underlying disease and delaying disease progression; however, their efficacy in preventing or reversing fibrosis is limited (Abbad, Esteve & Chatziantoniou, 2025). Although key profibrotic pathways such as transforming growth factor-β (TGF-β) pathway, have been identified as important targets, their multifaceted effects pose substantial clinical challenges, making the development of safe and effective antifibrotic therapies extremely difficult (Hong et al., 2025).

To overcome this deadlock, researchers must look beyond the genome to the epigenome. Epigenetic regulation acts as a molecular bridge that translates environmental stress into the cellular memory of fibrosis. Emerging evidence has confirmed that epigenetic changes are the central drivers of renal fibrosis (Zhao et al., 2023). Recent studies have confirmed that epigenetic regulatory mechanisms such as DNA methylation (Yan et al., 2024b), histone modification (Li et al., 2024), and RNA modification (Wang et al., 2022b) play a central role in this irreversible process. They are not only a key bridge connecting genes and the environment but also a crucial switch that determines the initiation and persistence of the fibrosis program. Therefore, epigenetic regulation integrates genetic risks and environmental signals, profoundly influencing the expression of the fibrosis gene network and is a core determinant of CKD progression. Targeting these reversible epigenetic abnormalities is a highly promising strategy for developing novel therapies that can precisely block or reverse renal fibrosis.

This review aims to systematically integrate the latest research on epigenetic mechanisms such as DNA methylation, histone modification, and noncoding RNA regulation, in renal fibrosis, clarify the core regulatory network, and ultimately guide the development of diagnostic markers and targeted therapeutic strategies, to provide ideas for developing precise antifibrotic therapies based on epigenetic regulation.

## Survey Methodology

To conduct a comprehensive and systematic literature review on the role of epigenetics in alleviating renal fibrosis, we conducted a thorough search across multiple databases, including PubMed (https://pubmed.ncbi.nlm.nih.gov/) and the Web of Science (https://www.webofscience.com/). Our search strategy included Medical Subject Headings and free-text keywords such as “renal fibrosis/CKD + epigenetics/DNA methylation/histone modification/noncoding RNA + therapy/biomarker” to maximize search sensitivity. During the screening process, specific exclusion criteria were applied to ensure the relevance and quality of the data included in this review. Studies on non-renal fibrosis, case reports, comments, and letters were excluded. The search results were carefully screened, and only the most relevant and updated articles were included. This process involved removing duplicate content, assessing the availability of full texts, and prioritizing literature with significant impact factors.

## Epigenetic Characteristic Map of Renal Fibrosis

Epigenetic regulation is the stable but reversible control of gene activity without changing the DNA sequence. Epigenetic regulation can be described by four distinct actions: adding chemical marks, identifying marks, removing marks, and changing the position of nucleosomes. These regulatory actions transform the same DNA sequence into active, paused, or repressed chromatin states (Allis & Jenuwein, 2016). Kidney fibrosis helps injured tubular cells, fibroblasts, endothelial cells, immune cells, and podocytes retain memory of prior stress. This memory can be harmful if it keeps fibrotic genes active after an initial injury. It can also exert protective effects by preserving repair programs, antioxidant genes, or anti-inflammatory pathways. Therefore, epigenetic mechanisms should not only be viewed as disease drivers. These are regulatory layers that can either worsen or limit fibrosis, depending on the cell type, timing, and target genes. In renal fibrosis, damaged kidney cells do not merely respond by activating isolated genes.

**Figure 1:**
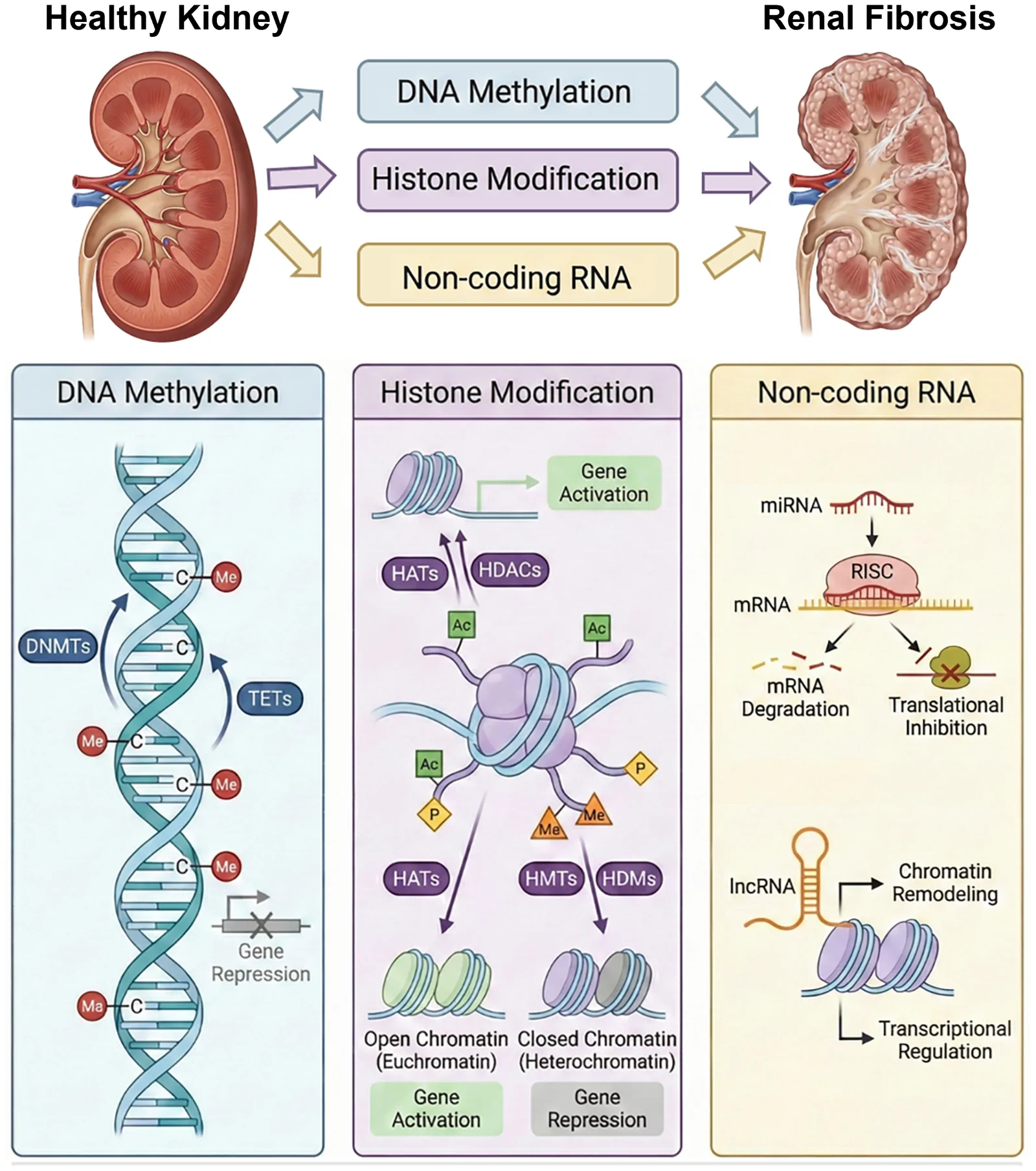
Epigenetic regulatory networks in renal fibrosis . The core epigenetic regulatory network underlying renal fibrosis is composed of three key modules: DNA methylation (mediated by DNMTs/TETs), histone modifications (acetylation/methylation by HATs/HDACs/HMTs/HDMs), and non-coding RNAs (miRNAs/lncRNAs) jointly shape chromatin remodeling and determine the activation or repression of fibrosis-related genes. (Abbreviations: Ac, Acetyl Group; C, Cytosine; HATs, Histone Acetyltransferases; HDMs, Histone Demethylases; Me, Methyl Group; P, Phosphate Group; RISC, RNA-induced Silencing Complex; TETs, Ten-eleven translocation enzymes).

During renal fibrosis, multilevel epigenetic reprogramming occurs, including dynamic changes in DNA methylation, histone modifications, and the noncoding RNA network, which collectively constite the characteristic molecular profile of disease progression (Fig. 1).

### DNA methylation

DNA methylation is primarily mediated by DNA methyltransferases (DNMTs). It often represses transcription when present in promoter CpG islands. DNA methylation is a fundamental mechanism that controls gene activity (Moore, Le & Fan, 2013). This method involves adding a tiny chemical tag, known as a methyl group, directly to the DNA ladder. This addition usually occurs at specific locations where a cytosine base is adjacent to a guanine base. When methyl groups accumulate near the beginning of a gene, they physically block the machinery required to read the gene. High levels of methylation typically silence gene expression. Genome-wide DNA methylation analysis revealed significant methylation abnormalities in a kidney fibrosis model. Experimental evidence has confirmed that the overall DNA methylation level in fibrotic kidneys is elevated and that the expression of DNA methyltransferases (DNMTs) is induced (Xiao et al., 2024).

The methylation status of key genes is directly related to the fibrotic phenotype; for example, hypermethylation of the miR-219a-2 promoter is associated with reduced miR-219a-2 expression and activation of downstream profibrotic signaling, suggesting that methylation-dependent micro RNA (miRNA) silencing may contribute to ECM accumulation (Wei et al., 2025). Similar evidence has been reported for N-Myc downstream-regulated gene 2 (NDRG2), which is reduced in fibrotic kidneys in association with promoter hypermethylation. Restoration of NDRG2 expression inhibits fibroblast activation, indicating that the loss of this methylation-sensitive gene may remove an important antifibrotic brake (Zhao et al., 2023). In addition, UHRF1, a key cofactor for DNMT1-mediated maintenance of DNA methylation, is upregulated in activated renal fibroblasts, and its absence inhibits DNA methylation at fibrosis-related genes (Gu et al., 2025). Taken together, these findings suggest that DNA methylation contributes to renal fibrosis by reshaping specific gene networks. However, most evidence remains preclinical, and future research should clarify whether these methylation changes are causal drivers or adaptive responses.

### Histone modification

Histone modifications alter how tightly the DNA is packed around histones. Histone modifications represent a critical epigenetic mechanism that dynamically regulates chromatin architecture and transcriptional accessibility. These covalent modifications occur primarily on the N-terminal tails of histone proteins and include acetylation, methylation, phosphorylation, ubiquitination, and sumoylation. The combination of these modifications determine the condensed state of DNA bound to nucleosomes. Histone modifying enzymes exhibit spatiotemporally specific expression patterns in fibrotic kidneys. Chromatin immunoprecipitation sequencing (ChIP-seq) studies have shown that histone acetyltransferase p300 is significantly upregulated in patients with focal segmental glomerulosclerosis and in various animal models of renal fibrosis, and that accelerates disease progression by regulating the transcription of pro-fibrotic genes. This finding suggests that histone acetylation may regulate fibrosis not only within tubular cells but also through paracrine crosstalk with endothelial cells (Kim et al., 2025). Histone methylation also shows strong regulatory potential. SETD2, as the sole catalytic enzyme for histone H3K36 trimethylation (H3K36me3), and its absence mediates epigenetic regulation, which can promote renal fibrosis (Liu et al., 2023a). In diabetic kidney disease (DKD), altered methylation of H3K4, H3K9, H3K27, and H3K36 affects several renal cell types. It is linked to podocyte injury, tubular epithelial injury, fibroblast activation, mesangial expansion, endothelial dysfunction, and ECM deposition (Qu et al., 2024). Additionally, a study has shown that the natural product epimedium glycoside A (GNA) inhibits TGF-β1-induced mesenchymal transformation in renal tubular epithelial cells by regulating histone methylation, highlighting the pathological regulatory value of modifying enzymes (Tao et al., 2022). Taken together, these studies demonstrate that histone modifications are not merely markers of fibrotic injury. They can also influence the intensity, duration, and specificity of fibrotic signaling

### Non-coding RNA

Traditionally, RNA has been viewed as a messenger that carries DNA instructions to build proteins. However, many RNAs do not encode proteins. These noncoding RNAs (ncRNAs) act as crucial regulatory managers in cells. They vary in size and have different functions. ncRNAs include microRNAs (miRNAs), long non-coding RNAs (lncRNAs), and circular RNAs. MiRNAs usually repress their target mRNAs. LncRNAs can act as scaffolds, guides, sponges, or chromatin regulators. These RNAs connect DNA methylation, histone marks, and classical signaling pathways. The reciprocal relationship between miRNAs and epigenetic enzymes in human diseases has been widely described (Piletic & Kunej, 2016). In renal fibrosis, ncRNAs often shape disease progression by forming regulatory networks centered on the TGF-β, Wnt/β-catenin, mitogen-activated protein kinase (MAPK), and ECM pathways. At the miRNA level, methylation-dependent silencing of miR-219a-2 may remove the antifibrotic brake, thereby promoting profibrotic signaling (Wei et al., 2025). In DKD, miR-342-3p expression is reduced, whereas that of its target SRY-box transcription factor 6 (SOX6) was increased. Restoring miR-342-3p reduced high glucose-induced TGF-β1, fibronectin, and collagen IV expression in mesangial cells (Jiang et al., 2020). In lupus nephritis, urinary exosomal levels of miR-21, miR-150, and miR-29c correlate with renal chronicity and may help predict fibrosis progression (Solé et al., 2019). Similarly, lncRNAs influence renal fibrosis by interacting with chromatin-modifying complexes, regulating transcription, splicing, RNA stability, and translation of fibrosis-related genes. (Tan et al., 2024). For example, lncRNAs can interact with chromatin remodeling complexes and alter the transcriptional activity of profibrotic genes, thereby exerting antifibrotic effects in various diseases (Ge et al., 2019; Guan, Sun & Zhang, 2022; Wang et al., 2020). Overall, these ncRNAs form a complex pathological regulatory network by targeting key signaling molecules (Xue et al., 2021; Zhao et al., 2021). Future studies should clarify which ncRNAs are causal in human CKD and can be safely targeted. Notable limitations and unresolved controversies persist in the current field of ncRNA-epigenetic regulatory networks in renal fibrosis. The vast majority of mechanistic conclusions are derived from *in vitro* cell assays and rodent preclinical models. In contrast, large-sample, long-term, longitudinal clinical evidence in human CKD remains insufficient and heterogeneous. Moreover, systemic interventions targeting ncRNA pathways inevitably disrupt global epigenetic homeostasis, leading to severe off-target epigenetic effects, unpredictable long-term organ toxicity, and unsatisfactory renal tissue-targeting efficiency. At present, definitive causality between ncRNA dysregulation and human renal fibrosis progression has not been rigorously validated in clinical settings, which severely restricts the clinical translation of ncRNA signatures into reliable diagnostic biomarkers and safe epigenetic therapeutic strategies.

## Research Progress on Key Epigenetic Regulatory Factors

### Bidirectional regulatory role of DNA methyltransferases

To understand the progression of renal fibrosis, researchers must study the specific enzymes that edit the epigenetic code. DNMTs are primarily responsible for gene reprogramming. DNMT expression is significantly increased in mouse models of kidney fibrosis. This pathological state is reversible, and the DNMT inhibitor 5-Aza can effectively erase these marks and alleviate the fibrotic phenotype (Xiao et al., 2024). The NDRG2 gene, an antifibrotic factor, has a highly methylated promoter region, which is the direct result of overexpression of DNMT1/3A/3B, leading to the inhibition of NDRG2 transcription and promoting fibrosis. The methyltransferase inhibitor 5-Aza can reverse the methylation of the NDRG2 promoter, restore its expression and alleviate fibrotic damage (Zhao et al., 2023). Furthermore, DNMTs rely on the co-factor UHRF1, which is upregulated in activated renal fibroblasts and acts as a critical guide. UHRF1 recruits DNMT1 to KLF15, creating a specific “UHRF1-DNMT1-KLF15” axis that silences this major transcriptional repressor of fibrosis (Gu et al., 2025). These findings reveal that DNMTs reprogram specific gene methylation and bidirectionally regulate the molecular essence of profibrotic and antifibrotic signaling networks.

### Profibrotic mechanism of histone deacetylases

DNA methylation locks down genes, whereas histone deacetylases (HDACs) inhibit transcription by altering chromatin structure, thereby promoting fibrosis. In DKD, HDAC2 exhibits significantly increased activity, which exacerbates tubular cell injury induced by high glucose and lipids. However, this can be countered by the sulforaphane (SFN), which inhibits HDAC2 and epigenetically upregulates the protective protein BMP-7 (Kong et al., 2021). HDAC3 is specifically induced in CKD tissues, where it promotes pathological damage by regulating the transition from inflammation to fibrosis (Wang et al., 2024b). The cross-dialogue mechanism between HDAC2 and DNMT1 identified in liver fibrosis research suggests that they may amplify fibrosis signals in the kidney *via* a similar epigenetic synergistic mechanism (Zhang et al., 2021a). In contrast, HDAC9 operates *via* a unique mechanism involving cell cycle control. Its expression is significantly upregulated in models like aristolochic acid nephropathy, where it forces renal tubular cells into G2/M phase arrest—a known cellular trigger for collagen production—making it a potent therapeutic target (Luo et al., 2022; Zhang et al., 2023b). Collectively, these findings indicate that HDACs govern fibrosis-associated transcriptional networks by disrupting the delicate balance of histone acetylation. Because different HDAC subtypes have distinct target sites and are expressed in different cell types, the research results for a specific HDAC cannot be generalized to all HDACs. Therefore, selective inhibitors and renal-targeted delivery systems are more reliable than broad-spectrum systemic HDAC inhibitors.

### Targeted intervention value of the MiRNA-mRNA interaction network

In the non-coding RNA regulatory network, the miRNA-mRNA interaction axis plays a significant role in the pathological remodeling of renal fibrosis. miR-219a-2 is directly regulated by DNA methylation in fibrotic kidneys, and the high methylation status of its promoter region has been verified by pyrosequencing to be positively correlated with disease progression, revealing a cross-level epigenetic dialogue between DNA methylation and miRNAs (Wei et al., 2025). Therapeutic strategies targeting miRNAs include LNA-modified antagonists that block the function of profibrotic miRNAs and nanocarriers delivering miRNA mimics to restore inhibitory miRNA activity against fibrosis; both have shown therapeutic potential in various kidney disease models (Chao, Kuo & Lin, 2024). However, each miRNA can regulate many transcripts, and multiple miRNAs can regulate each transcript. This pleiotropy creates an off-target risk. Delivery, durability, and immunogenicity are the major barriers.

### Protective roles of epigenetic regulation

Epigenetic mechanisms promote CKD progression but also help cells maintain their identity, control inflammation, and recover from injury. For example, methylation can prevent the abnormal activation of transposable elements and inhibit excessive inflammation (Dhillon et al., 2023). SETD2-dependent H3K36me3 can support Smad7 expression and restrain TGF-beta signaling (Liu et al., 2023a). miR-29 can reduce the expression of ECM genes (Solé et al., 2019). Glutathione peroxidase 4 (GPX4) preservation reduces ferroptosis-associated fibrosis downstream of Smad3 (Liu et al., 2025b). These examples demonstrate that broad systemic epigenetic inhibition may be unsafe. Precise therapy should remove harmful epigenetic memories while preserving protective repair programs. The dual roles of epigenetic mechanisms in renal fibrosis are shown in Table 1.

## Core Signaling Pathways of Epigenetic Regulation

Renal fibrosis is not driven by a single pathway but by a combination of signals involving the abnormal activation of multiple key regulatory networks. Although classical pathways like TGF-β and Wnt/β-catenin provide the instructions, epigenetic mechanisms regulate the intensity and magnitude of these responses, profoundly influencing the fibrotic process through synergistic interactions. This section focuses on the collaborative effects of these pathways with epigenetic modifications, as well as the epigenetic switch mechanism that drives the critical transformation from inflammation to fibrosis.

**Table 1:** Harmful and protective roles of epigenetic mechanisms in renal fibrosis.

**Epigenetic mechanism**	**Pro-fibrotic example**	**Source**	**Protective example**	**Source**
DNA methylation	The NDRG2 and HOXA5 genes are inactivated due to high methylation, which leads to renal fibrosis.	Xiao et al., 2024; Zhao et al., 2023	Methylation can suppress transposable elements and restrain excessive inflammation.	Dhillon et al., 2023
Histone modification	HDAC9 can promote inflammation or tubular G2/M arrest.	Zhang et al., 2023b	SETD2-mediated H3K36me3 supports Smad7 and helps restrain TGF-beta signaling.	Liu et al., 2023a
Non-coding RNAs	Erbb4-IR and LRN9884 promote fibrosis.	He et al., 2024	miR-29 family members can repress extracellular matrix genes.	Huang et al., 2025

### TGF-β/smad pathway

TGF-β is a central regulator of renal fibrosis. Downstream Smad signaling, especially Smad3, controls the expression of many fibrosis-related genes through both direct transcriptional binding and epigenetic remodeling. For example, Smad3 and enhancer of zeste homolog 2 (EZH2) can form a complex at fibrotic gene loci and increase the expression of fibroblast activation genes (Davis et al., 2022). TGF-β receptor (TGFBR) activation is also more complex than simple ligand binding. Active TGF-β can be released from latent extracellular complexes by thrombospondins, integrins, matrix metalloproteinases (MMPs), reactive oxygen species (ROS), and receptor transactivation pathways (Chia et al., 2024). This layered activation explains why broad upstream TGF-β blockade may show weak clinical translation, whereas downstream or context-specific targeting may be more useful. A study has shown that the preventive and therapeutic use of the TGF-β receptor 1 inhibitor TK-850 can alleviate fibrotic damage in mice with unilateral ureteral obstruction (UUO). Moreover, it has no adverse effects on body weight or survival rate in mice and thus has potential clinical therapeutic value (Palfrey et al., 2025).

Recent studies have identified two important mechanisms in this pathway. First, Smad3 represses GPX4, a key inhibitor of ferroptosis. In CKD biopsy samples and UUO models, high phospho-Smad3 levels were associated with low GPX4 levels and elevated ferroptosis markers. Smad3 deletion protects mice and cells from GPX4 loss, ferroptosis, and fibrosis, whereas GPX4 silencing restores the fibrotic phenotype (Liu et al., 2025b). These findings linked epigenetic transcriptional control to oxidative lipid injury and matrix deposition.

Recently, Smad3-targeted therapy has gained preclinical support. In db/db mice, the Smad3 inhibitor, SIS3, reduced renal fibrosis and inflammation. Early treatment at the prediabetic stage also reduced hyperglycemia, protected islet beta cells, and preserved paired box 6 (Pax6) expression, whereas late treatment mainly improved nephropathy rather than diabetes. Mechanistically, SIS3 restored the Smad3/Smad7 balance, suppressed the lncRNAs Erbb4-IR and LRNA9884, and reduced NF-κB-driven renal inflammation (He et al., 2024) These data support a more precise strategy that targets Smad3-dependent injury rather than blocking all TGF-β activity.

Conversely, the loss of protective epigenetic marks can unleash this pathway; for example, the downregulation of SETD2 leads to a decrease in H3K36me3 at the Smad7 locus, silencing this natural inhibitor and allowing TGF-β signaling to run unchecked (Liu et al., 2023a).

Histone deacetylases also join this network, with HDAC2 exacerbating diabetic renal fibrosis by inhibiting the anti-fibrotic factor BMP-7 (Yang et al., 2024), whereas inhibiting EZH2 with compounds like GNA can block the TGF-β/Smad3 axis by upregulating Smad7 (Srinivasan et al., 2022).

### Wnt/β-catenin pathway

The Wnt/β-catenin pathway is significantly activated in renal fibrosis, and multiple epigenetic mechanisms regulate its activity. At the RNA level, the N6-methyladenosine (m6A) methyltransferase METTL3 stabilizes β-catenin mRNA through the insulin-like growth factor 2 mRNA-binding protein 3 (IGF2BP3) axis, thereby strengthening profibrotic transcription (Long et al., 2024; Ren et al., 2024). Simultaneously, histone modifications regulate this switch: Sirt1 protects against endothelial-mesenchymal transition (EndMT) by inhibiting Wnt signaling (Wang et al., 2025), while JMJD3 (H3K27me3 demethylase) is upregulated in fibrotic kidneys and promotes fibroblast activation by activating β-catenin (Cai et al., 2025; Yu et al., 2021). In podocyte injury, sirtuin 6 (Sirt6) protects podocytes by blocking the Wnt1/β-catenin/renin-angiotensin system (RAS) axis, and β-catenin inhibition reduces RAS components in experimental membranous nephropathy (Miao et al., 2024).

DNA methylation also forms a dual mechanism, in which the UHRF1/DNMT1 complex hypermethylates the promoter of KLF15, a negative regulator of Wnt, thereby indirectly enhancing pathway activity (Gu et al., 2025; Noack et al., 2012). Furthermore, there is extensive crosstalk between Wnt and TGF-β; CCN5 can synergistically inhibit them (Jin et al., 2024b; Liu et al., 2025c); or they can be co-activated by lncRNAs like MIR210HG, which upregulates HMGA2 to enrich targets of both pathways (Ma et al., 2021).

Recent microbiome studies have expanded the field of Wnt signaling. In rodent and human tubulointerstitial nephropathy samples, dysregulated microbial-derived tryptophan catabolites are associated with activation of the AhR-Wnt/β-catenin axis. Altered Enterococcus aldenensis and Lactobacillus acidipiscis abundances correlated with tryptophan metabolites and tubulointerstitial fibrosis severity. AhR knockdown reduced Wnt1, β-catenin, and Twist expression, and polyporusterone A improved fibrosis while partly normalizing the microbiota-metabolite profile (Liu et al., 2026a). These findings suggest that Wnt activity is influenced by microbial metabolism, chromatin, and RNA regulation.

### From inflammation to fibrosis

Epigenetic modifications act as molecular switches during the transition from inflammation to fibrosis. HDAC3 is expressed in fibrotic kidneys and promotes the release of inflammatory factors and fibroblast transformation *via* deacetylation (Wang et al., 2024b). This transition is further fueled by immune cells, as macrophage polarization toward the M1/M2 phenotype is accompanied by Wnt pathway activation, creating a self-sustaining loop of macrophage-dependent fibrosis (Tian et al., 2024).

A novel and dangerous mechanism involves the loss of SETD2-mediated H3K36me3, thereby activating endogenous retroviral elements and amplifying the renal inflammatory response. This finding is important because it links the chromatin state, innate immune activation, and fibrosis. This also explains why broad interference with chromatin enzymes may result in unexpected immune effects (Dhillon et al., 2023). In addition, DNMTs participate in this transformation through global DNA hypermethylation, a process that can be reversed by 5-Aza to alleviate inflammatory damage (Xiao et al., 2024). Furthermore, Smad3 can also drive inflammation by reducing Smad7 and by inducing lncRNAs such as LRNA9884, which promotes monocyte chemoattractant protein-1 (MCP-1)-dependent renal inflammation (He et al., 2024).

These studies demonstrate that the epigenetic network composed of histone modifications, DNA methylation, and non-coding RNAs integrates inflammatory signals and fibrosis pathways, thereby driving a transition to an irreversible pathological state.

## Gut Microbiota and the Kidney Epigenome

Beyond cell-intrinsic epigenetic and signaling networks, the gut microbiota acts as a critical external regulator that modulates the renal epigenome and drives fibrotic progression, forming a gut–kidney epigenetic axis. The gut microbiota should be considered a part of the renal fibrotic environment. Human data show that gut microbial signatures mirror CKD stage and circulating nephrotoxin levels. In one discovery and validation study, CKD-associated taxa correlated with indoxyl sulfate and p-cresyl sulfate, and some bacterial markers discriminated patients with CKD from controls better than the urine protein-to-creatinine ratio in the early stages (Wu et al., 2020). A recent review summarized how dysbiosis increases uremic toxins, reduces protective short-chain fatty acids (SCFAs), and alters tryptophan metabolite levels. These metabolites can influence TGF-β/Smad, NF-κB, AhR, Nrf2, and inflammatory pathway (Jin et al., 2026).

Microbiota signals intersect with epigenetics in several ways. SCFAs Inhibit HDACs. Indoxyl sulfate, p-cresyl sulfate, trimethylamine N-oxide, and tryptophan metabolites promote oxidative stress, AhR activation, and inflammatory transcription(Jin et al., 2026). Therefore, microbiota-targeted strategies, including diet, probiotics, prebiotics, synbiotics, adsorbents, natural products, and fecal microbiota transplantation (FMT), may indirectly influence fibrotic gene expression. The field still needs stronger clinical trials to establish these as antifibrotic therapies. These microbiota–epigenome interactions also provide novel targets for developing microbiota-directed epigenetic therapy.

## Development of Diagnostic Biomarkers

The field is moving away from invasive tissue biopsies toward “liquid biopsies” that detect circulating epigenetic markers, offering a non-invasive window into renal health. Recent advances in detection technologies have enabled the high-precision identification of these specific chemical traces in blood and urine. High-throughput techniques, such as whole-genome analysis and pyrosequencing can now pinpoint abnormal methylation patterns in fibrosis models; for instance, researchers have successfully detected hypermethylation in the promoter region of miRNA-219a-2 in fibrotic kidneys (Xiang et al., 2022). Methylation-specific PCR (MSP) provides further validation, verifying the hypermethylation of the SIRT3 promoter, which correlates negatively with fibrosis severity. In addition to DNA, urine-derived non-coding RNAs such as miR-185-5p serve as powerful biomarkers; their expression levels are significantly correlated with the degree of renal tubular atrophy and interstitial fibrosis, providing a robust basis for early diagnosis (Brandt et al., 2024).

The diagnostic power increases when data layers are combined, as multiomics integration strategies significantly enhance the ability to warn patients early. Researchers have developed a “Methylation Risk Score” (MRS) based on blood epigenomic data which successfully predicted the risk of CKD in 487 patients with type 2 diabetes (AUC = 0.72) (Marchiori et al., 2025). When integrated with standard clinical factors, the performance of this model improved dramatically, achieving a negative predictive value of 94.6% and highlighting its ability to accurately rule out low-risk populations (Yan et al., 2024a). Furthermore, by combining single-cell epigenomics with spatial anchoring, researchers can now construct a map of the renal epigenetic regulatory network, revealing dynamic changes in chromatin accessibility during fibrosis, and identifying precise targets for early intervention (Gisch et al., 2024; Scotto et al., 2021).

Single-cell multiomics allow researchers to locate epigenetic injuries in specific renal cell populations. A 2024 study integrated single-cell RNA sequencing (scRNA-seq), single-nucleus assay for transposase-accessible chromatin sequencing (snATAC-seq), human biopsy data, proteomics, chromatin immunoprecipitation sequencing (ChIP-seq), and spatial transcriptomics. TThis study found that proximal tubular cells in fibrotic kidneys had lower SOD1 and SOD2 expression, suggesting reduced antioxidant capacity. Nuclear factor I X (NFIX) has also been identified as a regulator of IFI27, an apoptosis-associated gene. IFI27 overexpression increases cleaved caspase-3, *α*-smooth muscle actin (*α*-SMA), and collagen deposition in UUO kidneys (Chen et al., 2024). This evidence provides epigenetic therapy with a clearer cellular map. Instead of treating the kidney as a single tissue block, future studies should determine which cells carry harmful chromatin marks, which transcription factors are maintained, and which targets can be safely corrected. The SOD1/SOD2 and NFIX-IFI27 findings also underscore why antioxidant and antiapoptotic programs should be evaluated alongside classical antifibrotic genes.

Finally, epigenetics offers a tool to measure the “biological age” of the kidney, as the “epigenetic clock” quantifies age-related drift in DNA methylation providing a novel metric for prognosis. Clinical analysis has validated this approach, confirming that the methylation level of HOXA5 in renal biopsy tissues is positively correlated with the degree of fibrosis, and that its downregulation is associated with poor prognosis (Yan et al., 2021). These findings support the notion that the epigenetic clock can serve as an independent biomarker for predicting fibrosis progression and treatment response (Yan et al., 2024b).

## Epigenetic Regulatory-Targeted Strategies in Renal Fibrosis

Currently, the epigenetic regulatory strategies for renal fibrosis mainly include DNMT inhibitors, HDAC inhibitors, miRNA mimics/antagonists, and natural products. This section focuses on regulatory mechanisms (Table 2).

**Table 2:** Epigenetic Therapeutic Strategies.

**Therapeutic class**	**Compound**	**Target**	**Mechanism/Result**	**Source**
**DNMT Inhibitor**	**Decitabine** **(5-Aza)**	DNA Methyltransferases	Reverses *NDRG2*; alleviates fibrosis	Jiang et al., 2025; Liu et al., 2025a; Xia et al., 2025
**HDAC Inhibitor**	**Sulforaphane (SFN)**	HDAC2	Upregulates *BMP-7*; protects renal injury	Kong et al., 2021
	**ACY1215**	HDAC6	Blocks TGF-β/Smad and NF-kB pathway; Block the process of fibrosis	Gu et al., 2025
**miRNA mimics/ antagonists**	**rAAV9**	miR-29b	Reduce renal tubular interstitial fibrosis and decrease collagen deposition	Switzer, 2026
**Natural Product**	**Dihydroartemisinin (DHA)**	DNMT1	Restores *Klotho* expression; alleviates fibrosis	Zhou et al., 2022
	**Gannoside A (GNA)**	EZH2-Smad7 Axis	Blocks TGF-β/Smad3 signaling; alleviate fibrosis injury	Huang et al., 2024a
	**polydatin, dihydromyricetin, baicalin, and Biochanin A**	regulate complex networks of histone modifications and non-coding RNAs	Correct metabolic disorders and fibrosis	Guo et al., 2026; Yan et al., 2023; Yang et al., 2021
**Combination Therapy**	**C02S (Dual- acting agent)**	DNMT + HDAC	Induces “viral mimicry”; enhances immunity	Huang et al., 2024b; Yang et al., 2024
**Clinical and translational evidence**	**SIS3**	Smad3/Smad7	Early treatment reduced hyperglycemia and renal fibrosis; later treatment mainly protected kidney.	He et al., 2024
	**UBCS039/ICG-001**	Sirt6-Wnt1/ubrk beta-catenin-RAS	Preserved podocyte markers and reduced Wnt/RAS signaling in membranous nephropathy models.	Miao et al., 2024
	**GPX4**	Smad3-GPX4- ferroptosis axis	Smad3 represses GPX4; preserving GPX4 may reduce ferroptosis- associated fibrosis.	Liu et al., 2025b
	**Probiotics, prebiotics, synbiotics, FMT, natural products**	Gut-kidney axis; AhR-Wnt/beta-catenin; SCFAs	Aims to reduce uremic toxins, restore protective metabolites, and modulate inflammatory/fibrotic signaling.	Jin et al., 2026; Liu et al., 2026a; Wu et al., 2020

### Clinical and translational evidence

The clinical translation of antifibrotic therapy remains difficult. Broad inhibition of TGF-β is a clear example. A phase 2 trial of anti-TGF-β1 antibody therapy in diabetic nephropathy did not show any convincing clinical benefit when added to standard care (Voelker et al., 2017). This result supports a more selective strategy, such as targeting Smad3, GPX4-dependent ferroptosis, cell-specific Wnt signaling, or epigenetic enzymes that are active in diseased cell populations. Currently, most epigenetic therapies for renal fibrosis are preclinical. The strongest near-term clinical value may come from biomarkers, patient stratification, and safer delivery systems, rather than the immediate use of systemic epigenetic drugs.

Clinical biomarker studies are more advanced than treatment trials. Blood-based methylation scores have been used to predict incident CKD in individuals with type 2 diabetes, and gut microbiota studies have linked microbial taxa to CKD stage and uremic toxins (Marchiori et al., 2025; Wu et al., 2020). These findings may help identify patients who may benefit from earlier mechanism-guided interventions. However, their clinical use requires validation in larger cohorts, standardized sample handling, and proof that biomarker-guided treatment improves outcomes.

### DNMT inhibitors

DNMT inhibitors have shown potential in the treatment of renal fibrosis. Studies have shown that global DNA methylation levels are elevated in mouse models of renal fibrosis and are associated with the induction of DNA methyltransferase expression. The clinical drug decitabine (a DNMT inhibitor) can effectively break this cycle. It functions by reversing the hypermethylation of the promoter regions of key genes (Jiang et al., 2025; Liu et al., 2025a; Xia et al., 2025). Additionally, the natural product dihydroartemisinin (DHA) has been shown to suppress renal fibrosis by directly targeting DNMT1, promoting its degradation, and thereby reversing hypermethylation of the *Klotho* gene promoter, restoring its expression. This mechanism alleviates fibrosis in mouse models and further confirms the clinical translational value of targeting DNMT in renal fibrosis (Zhou et al., 2022). Most promising is the potential for combination therapy: pairing DNMT inhibitors with HDAC inhibitors can trigger a “viral mimicry” response, activating endogenous retroviruses (ERVs) and interferon pathways to enhance the immune system’s clearance of fibrotic cells (Huang et al., 2024b; Yang et al., 2024). Therefore, the current data more strongly support DNMT pathways as mechanistic targets and biomarker candidates than long-term systemic DNMT inhibitor therapy in CKD.

### HDAC inhibitors

HDAC inhibitors (HDACi) exert their therapeutic effects by simultaneously dampening inflammation and blocking fibrosis; however, isoform specificity remains a challenge. Studies have identified HDAC3 as a critical driver that is upregulated in CKD models, and its deficiency or inhibition significantly reduces the expression of inflammatory cytokines, proving its value as a target (Jin et al., 2024a; Zhao et al., 2025). Similarly, HDAC6 promotes fibrosis specifically by activating the TGF-β/Smad and NF-κB signaling axes. Using the selective inhibitor ACY1215 or siRNA to silence HDAC6 effectively serves as a signaling link, preventing the transition to a fibrotic state (Gu, Liu & Lan, 2024). Other agents, such as SFN work *via* a protective mechanism by epigenetically upregulating bone morphogenetic protein-7 (BMP-7) to counteract diabetic renal injury (Kong et al., 2021). In combination with other anti-inflammatory treatments, HDACi can be used with DNA methylation inhibitors or anti-inflammatory drugs to produce a synergistic effect, for example, by enhancing the response to immune checkpoint therapy or inhibiting the inflammatory-fibrotic transformation (Lewis et al., 2024; Yang et al., 2024; Zheng et al., 2025b). However, the clinical applications of broad-spectrum HDAC inhibitors are currently limited by insufficient selectivity and systemic toxicity (Lin et al., 2025; Zheng et al., 2025b). Therefore, the most realistic approach involves isoform-selective inhibition combined with kidney- or cell-targeted delivery.

### miRNA mimics/antagonists

In non-coding RNA therapy, a delivery system for miRNA mimics or antagonists is crucial for enhancing therapeutic efficacy. Studies have shown that miRNA hydrogel delivery systems can load exogenous miRNA inhibitors or mimics, enabling targeted intervention in inflammatory diseases and effectively preventing miRNA degradation or inactivation caused by environmental stressors (Hu et al., 2024). Viral vectors offer various solutions. For instance, the delivery of miR-29b using a recombinant adeno-associated virus (rAAV9) vector has been shown to significantly alleviate renal tubulointerstitial fibrosis and reduce collagen deposition, demonstrating the high translational potential of this vector system (Switzer, 2026). Moreover, delivery systems targeting miRNA-1303 have made progress in cancer treatment, and their design strategies can provide a reference for the treatment of renal fibrosis (Zheng, Hu & Badehnoosh, 2025a). However, renal cell specificity, immune activation, durability, and off-target binding must be evaluated before clinical application. The lncRNA field adds another layer of opportunity, but most lncRNA studies are mechanistic and require independent validation in human samples.

### Natural products

Natural products are gaining attention as “epidrugs” because they often regulate multiple epigenetic targets simultaneously with lower toxicity than synthetic agents. For example, Gynostemma pentaphyllum aglycone acts as a histone modulator, specifically inhibiting the repressive H3K27me3 mark and downregulating profibrotic genes to alleviate injury in both UUO and folic acid-induced fibrosis models (Huang et al., 2024a). As mentioned previously, dihydroartemisinin functions as a DNA methylation eraser by targeting DNMT1, thereby restoring Klotho expression (Liu et al., 2025a). The library of active compounds is expanding with agents such as polydatin, dihydromyricetin, and baicalin, which regulate complex networks of histone modifications and noncoding RNAs to treat metabolic disorders and fibrosis (Yan et al., 2023; Yang et al., 2021). Biochanin A, a natural isoflavone, reduced renal fibrosis in a murine UUO model and in TGF-β1-stimulated tubular cells. This study showed a dose-dependent reduction in the expression of ECM genes and partial epithelial-mesenchymal transition markers. Mechanistically, biochanin A inhibits Smad2/3 phosphorylation and reduces Smad3 protein expression. Smad3 overexpression weakens the antifibrotic effect, supporting a Smad3-dependent mechanism (Guo et al., 2026). The multi-effectiveness of these natural products provides a vital new direction for drug development, offering a strategy to safely modulate the epigenetic landscape over the long term.

## Current Challenges and Controversies in Epigenetic Targeting of Renal Fibrosis

### Technical barriers to tissue-specific delivery

Clinical application of epigenetic therapy for the treatment of renal fibrosis faces significant challenges in terms of tissue targeting. Existing drug delivery systems are unable to achieve targeted enrichment of renal lesions, resulting in insufficient drug accumulation in damaged kidneys and thus affecting therapeutic efficacy (Fu et al., 2022; Gu, Liu & Lan, 2024). To compensate for this, traditional treatments often require higher doses; however, this brute-force approach frequently leads to dangerous systemic toxicity caused by low delivery efficiency (Huang et al., 2024a; Liu et al., 2026b). Although nanocarriers can enhance renal targeting (Fu et al., 2022; Gu, Liu & Lan, 2024), their design still requires the optimization of carrier materials, surface modification, and release kinetics to overcome biological barriers (Liu et al., 2023b). Additionally, myofibroblasts, the key cells in fibrosis, remain a target of specific targeting strategies that still rely on peptide modifications and other emerging technologies; however, stability and large-scale production remain bottlenecks (Cheng et al., 2022; Wu et al., 2021).

Second, the kidney contains many cell types that respond differently to the same epigenetic drugs. For example, proximal tubular cells may require in the restoration of antioxidant chromatin programs, whereas macrophages may require reduced Wnt secretion or inflammatory lncRNA activity. Single-cell epigenomics can guide this design, but delivery tools must still reach the correct cells without disturbing protective repair programs.

### Control of off-target effects of epigenetic drugs

The second major challenge is specificity, because epigenetic enzymes regulate the entire genome, and targeting them can cause widespread collateral damage. For instance, HDACi can induce widespread chromatin remodeling and alter the expression of thousands of genes across the genome (Frank et al., 2016). Research indicates that the transcriptional chaos induced by HDACi differs significantly from the targeted effects of genetic inactivation, suggesting complex, off-target mechanisms that remain unclear (Toro et al., 2023). Similarly, although small molecule inhibitors like the BET inhibitor JQ1 show efficacy in preclinical models, their lack of tissue selectivity means they may interfere with normal cell functions throughout the body (Saiz et al., 2024). Nanodeivery systems can reduce the risk of off-target effects by enhancing drug targeting (Li, Chen & Li, 2024), but how to precisely regulate the cell specificity of epigenetic editing tools remains a challenge (Zhang et al., 2021b; Zhou et al., 2023). In addition, noncoding RNA therapies must address the immunogenicity of delivery vectors for nontarget uptake tissue (Yan et al., 2021; Zhang et al., 2023a).

### Absence of long-term safety assessment standards

Finally, because a systematic evaluation system for the epigenetic drug treatment of chronic diseases such as CKD has not yet been established, researchers face a significant long-term safety issue. This critical gap is underscored by the recognized side effects and limitations of these agents, particularly in oncology where their use is well established. For instance, the mechanisms of nucleoside analog DNMT inhibitors involve the formation of DNA-drug adducts, which can induce a DNA damage response, highlighting a potential pathway to genomic instability that warrants careful evaluation in long-term treatment settings (Suraweera, O’Byrne & Richard, 2025). Still, there is a lack of long-term follow-up data for verification. Although epigenetic drugs can reverse fibrotic phenotypes in animal models, their potential effects on the reproductive system and developmental processes remain unclear (Talati & Gaikwad, 2025; Wang et al., 2024a). A core controversy in clinical translation is the “rebound effect”: it remains unclear whether the reversibility of epigenetic modifications leads to disease recurrence after drug withdrawal, or whether drugs might accidentally erase beneficial “epigenetic memory” (Saiz et al., 2024; Zhang et al., 2024). Currently, there are no specific safety guidelines for epigenetic kidney drugs, creating an urgent need to establish cross-species long-term toxicity models before their widespread clinical application.

## Future Perspectives and Research Directions

### Precise analysis of single-cell epigenomics

Epigenomic analysis at single-cell resolution is a key tool for revealing the cellular heterogeneity of renal fibrosis. By integrating single-nucleus RNA sequencing (snRNA-seq), chromatin accessibility (ATAC-seq), and multiple protein modification assays (H3K27ac, H3K4me1/3, H3K27me3), and a spatially anchored epigenomic map can be constructed to precisely analyze the dynamics of epigenetic reprogramming across different cell types in the fibrotic microenvironment. For example, in renal injury models, such techniques have identified fibrosis-related regulatory factors and their target gene networks (Chen, Chen & Zhang, 2022; Jin et al., 2024a). Moreover, the combination of whole-genome cytosine methylation and single-nucleus open chromatin analyses revealed a causal relationship between epigenetic variations and metabolic disorders in patients with CKD, providing a robust molecular basis for targeted intervention (Dang et al., 2022).

Recent single-cell multiomics studies have shown that proximal tubular cells lose their antioxidant transcriptional programs and acquire apoptosis-related pathways during fibrosis (Chen et al., 2024). Future studies should focus on snATAC-seq peaks, enhancer activity, RNA expression, spatial location, and functional validation. This approach can identify causal epigenetic nodes rather than descriptive markers.

### Development of programmable epigenetic editing tools

The future of therapy lies in editing the epigenome rather than inhibiting it, although epigenetic editors based on the CRISPR/Cas9 system currently require optimization of tissue targeting and editing specificity (Butterfield et al., 2025). The primary challenge remains the low delivery efficiency and off-target effects of current systems, necessitating the development of the kidney-specific vectors to precisely regulate profibrotic genes. For example, targeting the UHRF1-DNMT1 complex can reverse the hypermethylation of genes such as *Klotho*, but the *in vivo* delivery bottleneck must be resolved (Bao et al., 2024; Switzer, 2026). Additionally, editing histone-modifying enzymes to rebalance H3K27me3/H3K27ac ratios offers a strategy to block fibrosis-related signaling pathways like TGF-β/Smad3, but the long-term safety of such permanent modifications needs to be rigorously verified (Xia et al., 2025; Yu et al., 2021).

Epigenetic editing is conceptually different from conventional DNMT or HDAC inhibition. It uses a programmable DNA-binding domain, such as a zinc finger protein, transcription activator-like effector, or nuclease-dead CRISPR system, to bring an epigenetic writer or eraser to a chosen genomic locus. The goal is to rewrite the local epigenetic signature and alter gene expression without altering the DNA sequence. Recent clinical translations have begun outside of kidney disease; however, the main barriers remain target specificity, durable yet reversible reprogramming, immune response, and delivery (Heller, Bintu & Rots, 2026).

For renal fibrosis, epigenetic editing remains a future direction rather than a current treatment. In principle, it can reactivate protective genes, such as Klotho, NDRG2, HOXA5, or Smad7, or silence selected profibrotic loci in tubular cells, fibroblasts, macrophages, or podocytes. In practice, kidney-specific delivery, cell-specific promoters, transient editor exposure, and long-term safety testing are required before this strategy can be considered for CKD (Heller, Bintu & Rots, 2026)

### Application of organoid models in drug screening

To bridge the gap between animal models and clinical reality, human-derived kidney organoids are emerging as powerful tools for simulating complex fibrotic pathological phenotypes in high-throughput screening. For instance, kidney organoids treated with TGF-β1 successfully reproduced the process of myofibroblast differentiation, enabling researchers to identify Smad3-dependent fibrotic regulatory modules through chromatin conformation analysis (Liu et al., 2025b). When combined with single-cell multiomics techniques, these models allow for the real-time evaluation of how epigenetic inhibitors alter cell fate transformation, significantly accelerating the optimization of preclinical drugs (Mimura, Chen & Natarajan, 2025; Tao et al., 2022).

Organoid and *ex vivo* models should also include immune cells, fibroblasts, endothelial cells, and microbial metabolites when possible. This will help model the real fibrotic niche, where tubular injury, macrophage signals, Wnt/β-catenin activation, TGF-β/Smad3 signaling, and gut-derived metabolites interact.

### Development of small-molecule inhibitors targeting epigenetic regulation

As editing technologies mature, the immediate priority is developing highly selective small molecule inhibitors to overcome the off-target effects of existing drugs. The key directions are as follows:

- DNMT inhibitors: Optimizing the renal targeting of decitabine, which exerts antifibrotic effects by reversing the high methylation of gene promoters, such as NDRG2 and SIRT3, but reduces myelotoxicity (Molina-Cerrillo et al., 2022).

- HDAC inhibitors: Subtype-selective inhibitors can inhibit the transcription of pro-inflammatory genes, and reduces fibrosis by upregulating BMP-7 (Ma et al., 2016; Wang et al., 2022a).

- EZH2 inhibitor: ZLD1039 blocks YAP activation by inhibiting H3K27me3 modification and alleviating fibrosis (Xia et al., 2025), but its ability to enrich for renal tissue needs to be verified.

- Natural product derivatives: Such as Gannoside A (GNA), which regulates TGF-β signaling by inhibiting the EZH2-Smad7 axis; its epigenetic target specificity needs to be analyzed (Tao et al., 2022).

In the future, researchers must prioritize optimizing delivery systems, enhancing tissue targeting, and accelerating drug screening using organoid models to transform these findings into a clinical reality.

## Conclusion

In this review, we synthesized evidence indicating that the epigenetic regulatory network composed of DNA methylation, histone modifications, and noncoding RNAs is the core of renal fibrosis pathogenesis. Unlike traditional therapies that can slow fibrosis progression, targeting these reversible epigenetic modifications has transformative potential.

However, translating the prospects of laboratory research into clinical applications is hindered by numerous obstacles. The lack of tissue-specific delivery systems, poor cell selectivity, and potential risks, such as disruption of homeostasis, hinder current efforts. Additionally, most preclinical evidence comes from short-term rodent studies, which limit its scope of application. However, solving these problems requires long-term exploration.

To advance this field, it is necessary to shift single-cell multiomics research from descriptive studies to the functional validation of pathogenic epigenetic regulatory factors, rather than relying solely on correlational associations. Simultaneously, new-generation programmable epigenetic editors must be optimized for kidney-specificity and systematically evaluated for persistence, reversibility, and off-target effects at single-gene-locus resolution. Improvements in efficacy will also depend on combining epigenetic intervention with strategies targeting interrelated biological systems to achieve synergistic benefits beyond single-target approaches.

The integration of single-cell technology, programmable editors, human organ models, and multiomics biomarkers has created unprecedented opportunities for precise epigenetic medicine of renal fibrosis. Converting this vision into clinical reality requires sustained interdisciplinary collaboration among nephrology, epigenetics, bioengineering, and pharmacology, as well as targeted solutions to address delivery, specificity, and safety challenges. If these efforts succeed, these therapies will establish epigenetic therapy as a truly transformative strategy for treating renal fibrosis, bringing new hope to patients and providing a blueprint for the curative treatment of various chronic fibrotic diseases.

## Additional Information and Declarations

### Data Availability

The following information was supplied regarding data availability: This is a Literature Review.
